# Cardiovascular risk assessment by FRS and SCORE in Iranian adult population

**DOI:** 10.1186/s40200-017-0316-4

**Published:** 2017-08-23

**Authors:** Alipasha Meysamie, Fereshteh Salarvand, MirHojjat Khorasanizadeh, Reza Ghalehtaki, Mahsa Eskian, Saeed Ghodsi, Shirin Ghalehtaki, Mehrshad Abbasi, Koroush Etemad, Fereshteh Asgari, Alireza Esteghamati

**Affiliations:** 1 Department of Community and Preventive Medicine, Faculty of Medicine, School of Medicine, Tehran University of Medical Sciences, Tehran, Iran; 2 Department of Dermatology, Razi Hospital, Tehran University of Medical Sciences, Tehran, Iran; 3 School of Medicine, Tehran University of Medical Sciences, Tehran, Iran; 4 Radiation Oncology Research Center, Cancer Institute, Tehran University of Medical Sciences, Tehran, Iran; 5 School of Medicine, Ahvaz Jondishapour University of Medical Sciences, Ahvaz, Iran; 6 Endocrinology and Metabolism Research Center (EMRC), Vali-Asr Hospital, School of Medicine, Tehran University of Medical Sciences, Tehran, Iran; 7 Center for Disease Control, Ministry of Health, Tehran, Iran

**Keywords:** Risk factors, Risk assessment, Coronary heart disease, Cardiovascular disease, SCORE, FRS, Iran

## Abstract

**Background:**

Handling the growing epidemic of coronary heart disease in developing nations hinges on primary prevention, which logistically requires directing preventive interventions to those at the highest risk. Therefore, implementing cardiovascular risk assessment profiles is crucial to distinguish high risk groups who truly need extensive preventive measures. We aimed to draw a picture of the cardiovascular risk profiles in the Iranian adult population for the first time.

**Methods:**

Demographic, anthropometric, and laboratory data as well as blood pressure and smoking status of 3944 subjects participating in the 2011 national surveillance of risk factors for non-communicable diseases were used to calculate the mean estimated risk of coronary artery disease and the relative frequency of low-, medium- and high-risk subjects based on FRS and SCORE indices in general population as well as different age, sex, and residence subgroups.

**Results:**

The average 10-year risk of coronary artery disease (FRS) and 10-year risk of fatal coronary and cerebrovascular accidents (SCORE) in the 25 to 64 year-old population was 13.82 and 0.72 respectively. The relative frequency of the intermediate- and high- risk subjects was 25.8 and 22.6% based on FRS and 9.2 and 1.8% based on SCORE respectively. Average FRS and SCORE were significantly higher among men than women, but were not significantly different among urban and rural residents.

**Conclusions:**

A significant proportion of the Iranian population, based on FRS model, will be at moderate to high risk of coronary events in the next 10 years. Urgent preventive plans are needed at the national level.

## Background

Coronary heart disease (CHD) is characterized by presence of atherosclerosis in epicardial coronary arteries and subsequent disruption of myocardial blood flow, which according to the extent of obstruction and pace of its progression could result into myocardial infarction (MI) and death. CHD is one of the most prevalent causes of death in many communities. It is responsible for about one-third of deaths among adults older than 35 in the United States. In 2008, among non-communicable diseases (NCDs), CHD was the leading cause of death (48%) in low-middle income countries [1]. Cardiovascular diseases including acute myocardial infarction, unstable angina, sudden cardiac death, and stroke has been reported to have a high and rising burden in Iran and claim lives of men and women even in young ages [2]. CHD is one of the main causes of mortality and morbidity in Iranian population, and according to the available evidence, it is responsible for about 50% of total annual deaths [3]. Despite lack of accurate data, some evidences show that the prevalence of CHD in Iran is currently 21%, and is increasing each year [4]. While age-adjusted mortality rate of CHD is decreasing in developed countries, on average, this rate has increased by 20–45% in Iran [5].

The growing epidemic of CHD in Iran needs to be addressed urgently. Although there has been a major progress in therapeutic techniques for patients with symptomatic CHD, many initial events are fatal, and the treatment options are more palliative than curative in nature. Consequently, a truly decisive effect on the CHD epidemic will require primary prevention of incident disease, considering that most cardiovascular risk factors are modifiable. Measures such as life style modifications mainly involving diet and physical activity, taking medications like statins and aspirin, and angiographic or surgical interventions could moderate these risk factors [6].

Most studies emphasize on the benefits of risk factor modifying interventions, but consider these interventions to be cost-effective only in medium- to high-risk groups [7]. Widespread inclusion of such programs into care routines of healthcare centers and primary-care physician offices is associated with significant costs and requires rigorous measures. Therefore, the use of risk assessment models which provide the opportunity to direct the preventive measures to the at-risk population is unavoidable.

Aiming to identify the at-risk individuals, various global risk assessment profiles, derived from longitudinal cohort studies, have been optimized in the past 30 years. A well-known risk profile, recommended in clinical guidelines, is Framingham Risk Score (FRS), which predicts the 10-year risk of incident CHD – including fatal and non-fatal MI, unstable angina and stable anginas – based on the level of individual risk factors. Another model mostly adopted in western European countries is the Systematic coronary Risk Evaluation (SCORE) model, which predicts the 10-year risk of incident fatal atherosclerotic cardiovascular events, including myocardial and cerebral infarction [8]. These two risk assessment profiles have been validated in different geographic regions using reliable statistical methods [9].

Since in our country, Iran, there is no community-based evidence regarding the risk of CHD and fatal cardio- or cerebro-vascular events according to FRS or SCORE models at the national level, we aimed to assess the overal risk of CHD and fatal cardio- or cerebro-vascular events in 25–64 year-old Iranian subjects in this study. Knowing the 10-year risk of CHD and fatal cardio- or cerebro-vascular events is helpful in planning preventive measures for populations at the highest risk, especially in the resource-limited settings.

## Methods

### Study population

In the Surveillance of Risk Factors of Non-communicable Diseases (SuRFNCD) in 2011, a total of 11,867 Iranian individuals aged 6–70 years (excluding the nomadic tribes who are not covered by the Iranian postal service) were surveyed using Random complex sampling. Among the 25–64 years-old included subjects, 4759 individuals consented for blood sampling. After the re-evaluation of data and laboratory samples, and exclusion of pregnant subjects, ultimately 3944 individuals with complete demographic, laboratory and smoking status information entered the statistical analysis as the final sample. More information regarding the sampling method has been previously described elsewhere [10].

### Questionnaires, measurements and definitions

According to the Surveillance of Risk Factors of Non-communicable Diseases (SuRFNCD) international program, STEPS questionnaires of world health organization (WHO) were subjected to the participants in 6 sections including demographic information, diet, physical activity, tobacco smoking, history of hypertension and diabetes. Laboratory examination of blood samples was performed for variables such as high density lipoprotein (HDL), total serum cholestrol, triglyceride and glucose. All tests were performed using quality controlled commercial kits (Pars Azmun, Karaj, Iran) distributed by the center for disease control (CDC) reference laboratory. Inflexible measurement tapes and portable digital scales were respectively used to measure height and weight. Height and weight were measured with subjects standing without socks and shoes and dressed lightly. Blood pressure was measured using calibrated sphigmomanometer on three occasions spaced for 5 min, and the mean value was used. Waist circumference was measured mid-way around the direct line that connects the lower costal margin and anterior–superior iliac spine at the end of normal expiration, with arms extended and alligned with body.

### Statistical analysis

Weighting of the data was initially performed in order to describe the overall population regarding the stratified cluster sampling method being applied. After excluding duplicate, missing and outlier data, weighting was performed based on the 2011 official national population census and strata of provinces, residence (urban or rural), gender (male or female), and age groups (10-year categories 25–34, 35–44, 45–54, and 55–64). The minimum and maximum weights were 99,951 for 35–44 year old male indiviuals living in urban areas of Bushehr province and 315 for 55–64 year old female indivuals living in rural areas of Ilam province. The median weight was 6880. The total extrapolated population size was 42,287,987. Thereafter, the data were analysed using complex sample survey method opting SPSS version 20 for Windows. The 10-year risk of coronary heart diseases and the 10-year risk of fatal cardiovascular events were calculated using FRS and SCORE functions respectively. Then the subjects were categorized into low-, intermediate- and high-risk groups based on their FRS and SCORE risk values. The mean score and relative frequency of any given gender, residential and age strata in each risk group were calculated with 95% confidence interval. The detailed methods of calculation of FRS and SCORE have been explained elsewhere [8, 11].

### Research ethics and patient consent

This study was approved by institutional review board of the Center for Disease Control of Iranian Health Ministry. All steps of this study were conducted in accordance with the guidelines and standards laid down in the latest revision of the Declaration of Helsinki. For confidentiality reasons, patients’ names were not used in the study, and the allocated codes were used instead. Written informed consent was provided by all participants.

## Results

### The 10-year risk of CHD based on FRS

The mean age of studied subjects was 38.40 years (CI95% = 37.6–39.21). 49.7% were male and 68.8% were urban residents. Of the participants, 40.6% were in the 25–34, 28.5% in 35–44, 19.9% in 45–54, and finally 11% in the 55–64 years age group.

The mean 10-year risk of CHD based on FRS in the studied population was 13.82 (CI95% = 13.47–14.17). The relative frequency of low-, medium- and high-risk subjects in the studied population was 51.6% (49.9–53.3), 25.8% (24.1–27.7) and 22.6% (21.2–24.0), respectively (Table 1).

**Table 1:** Mean FRS and intensity of FRS in the 25–64 years-old Iranian population

	Standard Population	Mean FRS	FRS Intensity (%)					
			Low (<10%)		Intermediate (10–19.99)		High (> = 20%)	
		Mean (CI95%)	*N* (national estimate^a^)	% (CI95%)	*N* (national estimate)	% (CI95%)	*N* (national estimate)	% (CI95%)
Gender								
Men	21,052,600	15.95 (15.37–16.53)	464 (9.345)	44.3% (41.7–47.0)	313 (5.478)	26.0% (23.1–29.1)	697 (6.253)	29.7% (27.3–32.1)
Women	19,714,939	11.54 (11.19–11.88)	1114 (11.680)	59.4% (57.4–61.4)	726 (5.048)	25.7% (23.7–27.7)	611 (2.938)	14.9% (13.7–16.3)
*P* value		< 0.001		< 0.001		0.1		< 0.001
Residential area								
Urban	29,104,663	14.01 (13.56–14.47)	995 (15.209)	51.4% (49.3–53.5)	674 (7.600)	25.7% (23.6–27.9)	899 (6.786)	22.9% (21.2–24.7)
Rural	13,183,323	13.31 (12.59–14.03)	583 (5.816)	52.2% (48.6–55.7)	365 (2.926)	26.2% (22.8–30.0)	409 (2.405)	21.6% (18.9–24.6)
*P* value		0.11		0.10		0.12		0.11
Age Group								
25–34	17,185,017	5.55 (5.27–5.84)	1004 (15.050)	89.8% (87.2–92.0)	90 (1.571)	9.4% (7.3–11.9)	7 (0.129)	0.8% (0.3–1.9)
35–44	12,051,216	12.91 (12.18–13.64)	384 (4.744)	41.9% (37.7–46.1)	336 (5.061)	44.7% (40.3–49.1)	84 (1.525)	13.5% (10.5–17.0)
45–54	8,408,945	22.30 (21.32–23.28)	127 (1.021)	12.6% (10.2–15.5)	327 (2.976)	36.8% (32.4–41.5)	320 (4.083)	50.5% (46.0–55.0)
55–64	4,642,807	31.36 (30.24–32.47)	63 (0.208)	4.6% (3.4–6.1)	286 (0.918)	20.1% (17.1–23.3)	897 (3.453)	75.4% (72.1–78.4)
*P* value		< 0.001		< 0.001		0.001		< 0.001
Total	42,287,987	13.82 (13.47–14.17)	1578 (21.025)	51.6% (49.9–53.3)	1039 (10.527)	25.8% (24.1–27.7)	1308 (9.191)	22.6% (21.2–24.0)

^a^In millions

The mean Framingham Risk Score was significantly higher in men than women; 15.95 (15.37–16.53) for men vs. 11.54 (11.19–11.88) for women. There was no statistically significant difference in FRS between urban and rural residents (*P* = 0.1). The mean FRS increased significantly by increasing age to reach 31.36 in the 55–64 age group, compared with mean FRS of 5.55 in the 25–34 age group.

The frequency of high-risk subjects in men was significantly higher than women, with 29.7% (27.3–32.1) for men vs. 14.9% (13.7–16.3) for women. However, there was no significant difference between urban and rural residents. In higher age groups, the frequency of high-risk subjects was significantly higher than lower age groups; e.g. 75.4% (72.1–78.4) in the 55–64 age group, compared with 0.8% (0.3–1.9) in the 25–34 age group.

### The 10-year risk of fatal cardio- and cerebro-vascular events based on SCORE

The mean 10-year risk of fatal cardio- and cerebro-vascular events based on SCORE in the studied population was 0.72 (CI95% = 0.68–0.75). The frequency of low-risk, medium-risk and high-risk subjects in the studied population was 89.0% (88.0–89.9), 9.2% (8.3–10.2) and 1.8% (1.5–2.2) respectively (Table 2).

**Table 2:** Mean SCORE and SCORE intensity in 25–64 year-old Iranian population

	Standard Population	Mean Score	SCORE Intensity (%)					
			Low (<2%)		Intermediate (2–4.99)		High (> = 5%)	
			*N* (national estimate^a^)	% (CI95%)	*N* (national estimate)	% (CI95%)	*N* (national estimate)	% (CI95%)
Gender								
Men	21,052,600	1.05 (0.99–1.11)	910 (17.479)	83.0% (81.3–84.7)	425 (2.917)	13.9% (12.2–15.7)	138 (0.656)	3.1% (2.5–3.9)
Women	19,714,939	0.36 (0.33–0.38)	2209 (18.812)	95.4% (94.7–96.0)	221 (0.826)	4.2% (3.6–4.9)	25 (0.075)	0.4% (0.2–0.6)
*P* value		< 0.001		< 0.001		< 0.001		< 0.001
Residential area								
Urban	29,104,663	0.72 (0.68–0.77)	2003 (26.371)	89.0% (87.9–90.1)	450 (2.693)	9.1% (8.1–10.2)	118 (0.554)	1.9% (1.5–2.3)
Rural	13,183,323	0.69 (0.63–0.76)	1116 (9.920)	89.0% (86.7–90.9)	196 (1.050)	9.4% (7.6–11.7)	45 (0.177)	1.6% (1.0–2.6)
*P* value		0.09		0.10		0.22		0.11
Age Group								
25–34	17,185,017	0.04 (0.043–0.544)	1102 (16.764)	100%	0	0	0	0
35–44	12,051,216	0.33 (0.30–0.36)	804 (11.245)	98.9% (96.8–99.6)	4 (0.129)	1.1% (0.4–3.2)	0	0
45–54	8,408,945	1.29 (1.20–1.38)	678 (6.461)	80.2% (76.3–83.6)	86 (1.427)	17.7% (14.4–21.7)	11 (0.166)	2.1% (1.1–3.7)
55–64	4,642,807	3.12 (2.89–3.35)	535 (1.820)	39.8% (36.0–43.8)	556 (2.186)	47.8% (43.6–52.0)	152 (0.565)	12.4% (10.2–15.0)
*P* value		< 0.001		< 0.001		< 0.001		< 0.001
Total	42,287,987	0.72 (0.68–0.75)	3119 (36.291)	89.0% (88.0–89.9)	646 (3.744)	9.2% (8.3–10.2)	163 (0.731)	1.8% (1.5–2.2)

^a^In millions

The mean score calculated based on SCORE was significantly higher in men than women; with 1.05 (0.99–1.11) for men vs. 0.36 (0.33–0.38) for women. There was no statistically significant difference in SCORE between urban and rural residents (*P* = 0.09). The mean SCORE increased significantly by increasing age to reach 3.12 (2.89–3.35) in the 55–64 age group, compared with mean SCORE of 0.04 (0.043–0.0544) in the 25–34 age group.

The frequency of high-risk subjects in men was significantly higher than women, with 3.1% (2.5–3.9) for men vs. 0.4% (0.2–0.6) for women. However, there was no significant difference between urban and rural residents. In higher age groups, the frequency of high-risk subjects was significantly higher than lower age groups; e.g. 12.4% (10.2–15.0) in the 55–64 age group, compared with 0% in the 25–34 age group.

## Discussion

The main goal of this study was to perform a cardiovascular risk assessment of the Iranian adult population. In order to achieve this goal, the 10-year risk of incident coronary artery disease based on Framingham Risk Score (FRS) and the 10-year risk of incident fatal atherosclerotic events including coronary artery disease and cerebrovascular accidents based on Systematic COronary Risk Evaluation (SCORE) were calculated in the 25–64 years-old population.

As was thoroughly explained in the Results section, mean FRS in Iranian population is considerably high (13.82), and more than 20% of the Iranian population which equals to 13.08 million people are in the high-risk group (FRS ≥ 20) for coronary artery disease. FRS was higher in older age groups, and in men compared with women. In contrast to expectations, there was no significant difference in mean FRS and frequency of high-risk individuals between urban and rural residents. Previously, the discrimination power of FRS for prediction of cardiovascular mortality at 5 and 10 years have been shown in Iranian urban population in Tehran Lipid and Glucose prospective study [12, 13].

The relatively high mean FRS has been pointed out earlier by other authors in specific Iranian populations. Among these examples are the mean FRS figures reported as high as 10.2 ± 7.1 in postmenopausal women by Eshtiaghi et al. [14], and 13.0 ± 8 in men and women with at least one cardiovascular risk factor by Nematy et al. [15].

In the study of Ford et al. in United States, the relative frequency of low-, medium- and high-risk groups based on FRS was 81.7, 15.5 and 2.9% respectively [16]. In this study, the mean FRS was higher in older age groups, and in men compared with women; as the relative frequency of high- and medium-risk subjects were respectively 5.3 and 38.7% in men and 0.9 and 4.3% in women. Although this general pattern is consistent with the results of our study, but in our study the frequency of medium- and high-risk subjects were much higher in the 25–64 year-old Iranian population. One reason could be the difference between study populations. Regarding risk assessment, best results can be achieved when the population being evaluated is similar to the reference population of the applied risk index. The reference population for FRS index was socio-economically advantaged Caucasian subjects; therefore this index over-estimates the risk in African, Native American and Asian ethnic groups [17]. In agreement with this statement, in a systematic review by Brindle et al., the predicted to observed ratios based on FRS ranged from an underprediction of 0.43 (95% CI 0.27 to 0.67) in a high-risk population to an overprediction of 2.87 (95% CI 1.91 to 4.31) in a lower-risk population, suggesting that the performance of FRS varies considerably between populations [18]. In another study, Chia et all showed that cardiovascular disease (CVD) risk in a multiethinic east Asian society calculated by FRS model could only make a moderate discrimination, with an area under the receiver operating characteristic curve (AUC) of 0.63 [19]. On this basis, it seems that using a model derived from characteristics of the Iranian population could predict the 10-year risk of coronary artery disease with more accuracy. But, until then FRS may act as an alternative risk assessment tool.

The mean SCORE index in Iranian population is relatively low (0.72), and based on this index about 2% of the total 25–64 year old population which equals to 730,000 people are categorized in the high-risk group (SCORE ≥ 5). As for FRS, the mean SCORE was higher in older age groups, and in men compared with women, and there was no significant difference in mean SCORE and frequency of high-risk individuals between urban and rural residents.

Our 10 year risk estimates of fatal cardio- or cerebrovascular events calculated using SCORE model were far lower than those of europian countries. One explanation for this difference is that we used this model for all above 25 year old population, whereas SCORE model was developed according to the mortality data of 40–75 year old population in mostly western Europian countries [8]. Interestingly, in our study, the prevalence of high risk subjects was 5.6 and 12.4% among over 45 and 55 year old population, respectively. There are no reports of risk stratification using SCORE in middle-eastern countries to make comparisons.

In comparison between FRS and SCORE models, Barroso et al. showed that among middle to old age Spanish population SCORE performed better in predicting CVD risk discrimination. In that study, 18.3 and 9.2% of the population were put into high risk category based on FRS and SCORE, respectively [20]. The lower estimates calculated by SCORE compared to FRS in our study and others in part returns to the aim of these models; SCORE was developed to predict only fatal events while the mission of FRS was to predict both non fatal and fatal cardiovascular events. Aside from this explanation other studies have shown that FRS usually estimates a higher risk inferred by each individual compared with SCORE [21].

Among the results it was noted that FRS and SCORE tend to markedly increase with age. It seems that the most prominent rationale for this could be the augmented insulin resistance and mean blood preasure as age increases. Actually, these two factors contribute most to the positive correlation between age and cardiovascular risk [22].

Moreover, in this study we showed that not only the mean 10-year risk of coronary artery disease based on FRS and SCORE indices is significantly higher in men than in women, but also the frequency of high-risk subjects is significantly higher among men. This difference could originate from the effect of sex hormones and their considerable protective role in women, as in the study of Eshtiaghi et al. in women aged 20–76 years was shown that mean FRS in the post-menopausal group of participants was significantly higher that the pre-menopausal group (10.2 vs. 1.6) [14].

In contrary to our expectations, there was no significant difference in mean FRS and SCORE and frequency of medium- to high-risk subjects between urban and rural residents in this study. Assuming that urban and rural residents are much different in diet and physical activity habbits, one might speculate that rural residents face a lower risk compared with urban residents, which is not true based on our results. Actually, there was no significant difference between urban and rural residents in many of the cardiovascular risk factors incorporated in FRS and SCORE models both in our study (data not shown) and previous studies [23]. These findings indicate that future preventive interventions should equally cover both urban and rural population.

Calculating 10-year risk of cardiovascular diseases through well-known global cardiovascular risk assessment models including FRS and SCORE is widely performed all over the world and is recommended for all adults. However, there are some limitations to these models. For instance, FRS may under- or over-estimate the risk in certain populations, its 10-year scope is relatively short, it mostly focuses on myocardial infarction and coronary artery disease-attributed death, and ignores family history. Regarding SCORE, one of the most important limitations is ignoring triglyciride and fibrinogen levels, and also family history of early-onset cardiovascular disease [8]. Categorizing the subjects into low-risk (<10%), medium-risk (10–20%) and high-risk (>20%) groups could be biased by up to 37%, especially in women and young adults. In order to address these limitations, one of the proposed solutions is to use life-time risk assessment models, as a considerable proportion of subjects with low 10-year risk, have a life-time risk of more than 39% [24]. Moreover, in order to further categorize intermediate risk individuals, more novel risk factors such as Lp-PLA_2_, LDL-P, fibrinogen, Lp-a, small dense low density lipoprotein (LDL), plasminogen activator inhibitor (PAI-1), IL-6, CRP, coronary artery calcification (CAC) and carotide intima-media thickness can be used [25].

### Limitations

Considering the large population of this study, the probability of non-cooperation of participants in filling out the questionnaires and providing blood samples and anthropometric measurements were among the limitations of our study. Therefore, the most important limitation of this study is the high amount of missing data both during data collection and data entry into the statistical software. Actually the authors faced a lot of missing, duplicate and outlier values. To moderate the effect of this problem, the duplicate and outlier data were cleared as much as possible, and in the final statistical analysis only the cleared data were weighted. Another limitation that severely impacts our conclusions is our cross-sectional design of the study. In fact, we can not say for sure whether the high risk subjects discriminated by FRS and SCORE models will be affected by cardiovascular accidents in future or not.

## Conclusion

In this study we showed that mean FRS in the 25–64 years-old Iranian population is markedly high, and more than 20% of the population are in the high-risk group. The mean risk and frequency of high-risk subjects were lower based on the SCORE index. These results show that in the upcoming years the Iranian population is going to be in great risk for cardiovascular events, and more rigorous primary prevention programs are urgently needed at the national level. However, when comparing these risk values with those of other populations, it should be noted that differences between the studied population and the reference population of the risk assessment model in use, increase the error of measurement. Therefore we propose that a risk assessment model derived from the Iranian population characteristics be designed and validated in future studies, and its power in predicting the risk of cardiovascular events be compared with available models.

## Abbreviations

AUC: Area under curve
CAC: Coronary artery calcification
CDC: Center for disease control
CHD: Coronary heart disease
CVD: Cardiovascular disease
FRS: Framingham Risk Score
HDL: High density lipoprotein
LDL: Low density lipoprotein
MI: Myocardial Infarction
NCDs: Non-communicable diseases
SCORE: Systematic COronary Risk Evaluation
STEPS: STEPwise approach to Surveillance
SuRFNCD: Surveillance of Risk Factors of Non-communicable Diseases
